# Biosecurity survey in relation to the risk of HPAI outbreaks in backyard poultry holdings in Thimphu city area, Bhutan

**DOI:** 10.1186/s12917-017-1033-4

**Published:** 2017-04-21

**Authors:** Tenzin Tenzin, Chador Wangdi, Purna Bdr Rai

**Affiliations:** 1 Disease Prevention and Control Unit, National Centre for Animal Health, Department of Livestock, Thimphu, Bhutan; 2 Bhutan Agriculture and Food Regulatory Authority, Ministry of Agriculture & Forests, Thimphu, Bhutan; 3 Laboratory Service Unit, National Centre for Animal Health, Department of Livestock, Thimphu, Bhutan

**Keywords:** Backyard poultry holdings, Biosecurity, Knowledge, Attitudes, Practices, HPAI and H5N1 virus, Bhutan

## Abstract

**Background:**

A questionnaire survey was conducted to assess the biosecurity and other practices of backyard poultry holdings and knowledge and practices of poultry keepers following an outbreak of highly pathogenic avian influenza (H5N1) virus in poultry in Thimphu city area, Bhutan.

**Results:**

The study identified 62 backyard poultry holdings in 12 settlement areas, and the owners were subsequently interviewed. The birds are kept in a low-input low-output system, fed locally available scavenging feed base, and supplemented with food scraps and some grain. Although the birds are housed at night in a small coop to protect them against theft and predators, they are let loose during the day to scavenge in the homestead surroundings. This invariably results in mixing with other poultry birds within the settlement and wild birds, creating favorable conditions for disease spread within and between flocks. Moreover, the poultry keepers have a low level of knowledge and awareness related to the importance of biosecurity measures, as well as veterinary care of the birds and reporting systems. Of particular concern is that sick birds within backyard holdings may not be detected rapidly, resulting in silent spread of disease and increased risk of humans contacting the virus (e.g. HPAI) from infected poultry. Nevertheless, all the respondents have indicated that they know and practice hand washing using soap and water after handling poultry and poultry products, but rarely use face-masks and hand gloves while handling poultry or cleaning poultry house.

**Conclusions:**

This study highlights the importance of educating poultry keepers to improve the housing and management systems of poultry farming within the backyard holdings in the Thimphu city area in order to prevent future disease outbreaks.

## Background

Highly pathogenic avian influenza (HPAI), subtype H5N1, was first reported in Southeast Asia in late 2003, and then spread rapidly with outbreaks being reported in 63 countries across Asia, Europe, Africa, and the Middle-East [1]. Since the emergence of HPAI virus in poultry in 2003, there has been 856 laboratory-confirmed human cases officially reported to World Health Organization (WHO) from 16 countries, including 452 deaths up to 3 October 2016 [2]. The outbreaks have had serious economic impact to the affected countries, with millions of birds either killed by the disease or mandatory culled in an effort to limit the spread of virus [3, 4]. Although, different countries have implemented various strategies aimed at preventing and mitigating infection within poultry with varying degree of success, in some countries, the virus remains entrenched within poultry populations [1, 5]. One of the factors responsible for outbreaks and the persistence of the virus in domestic poultry populations is cited to be the widespread practice of small holder backyard poultry farming and associated live bird markets [5–7]. This is mainly because basic biosecurity measures are rarely implemented in backyard poultry farming systems allowing HPAI to circulate within poultry populations resulting in a perpetual virus source to other poultry flocks [5, 8, 9]. Therefore, one of the most effective forms of protection against HPAI and other poultry diseases is biosecurity, which is principally the implementation of measures to prevent the introduction of infectious agents into the farm/environment (bio-exclusion) or containment measures to prevent spread of infectious agents from exiting in the event of outbreaks (bio-containment) [9–12].

In Bhutan, the poultry farming system comprise of both commercial and backyard holdings but backyard farming is predominant in the country. The first outbreak of HPAI (H5N1) virus was reported in February 2010 in a backyard poultry holding in the southwest Bhutan, near the border with India [13, 14]. Since then, at least seven separate outbreaks of HPAI (H5N1) have been confirmed at 21 locations in six districts with outbreaks reported every year in 2011, 2012 and 2013. The most recent outbreak occurred on 03 April, 2015 in a backyard poultry holding in Thimphu city area (capital of Bhutan) [15]. To our knowledge, no studies have been conducted to understand the biosecurity practices of backyard poultry holdings in Thimphu or elsewhere in the country. Therefore, it is important to understand the types of backyard poultry holdings and biosecurity practices in farms for better preparedness planning. In this context, we conducted a rapid biosecurity survey among the backyard poultry holdings in Thimphu city, as a part of rapid risk assessment following an outbreak of HPAI in one of the backyard holdings in Thimphu.

The main objectives of this study were to (1) identify backyard poultry holdings in Thimphu city area that have potential risk of possible outbreaks in future, (2) generate baseline information about flock characteristics and assess basic biosecurity practices, and (3) understand poultry keepers’ knowledge in relation to poultry keeping and personal hygiene practices to prevent incursion and transmission of HPAI.

## Methods

### Study area

**Fig. 1:**
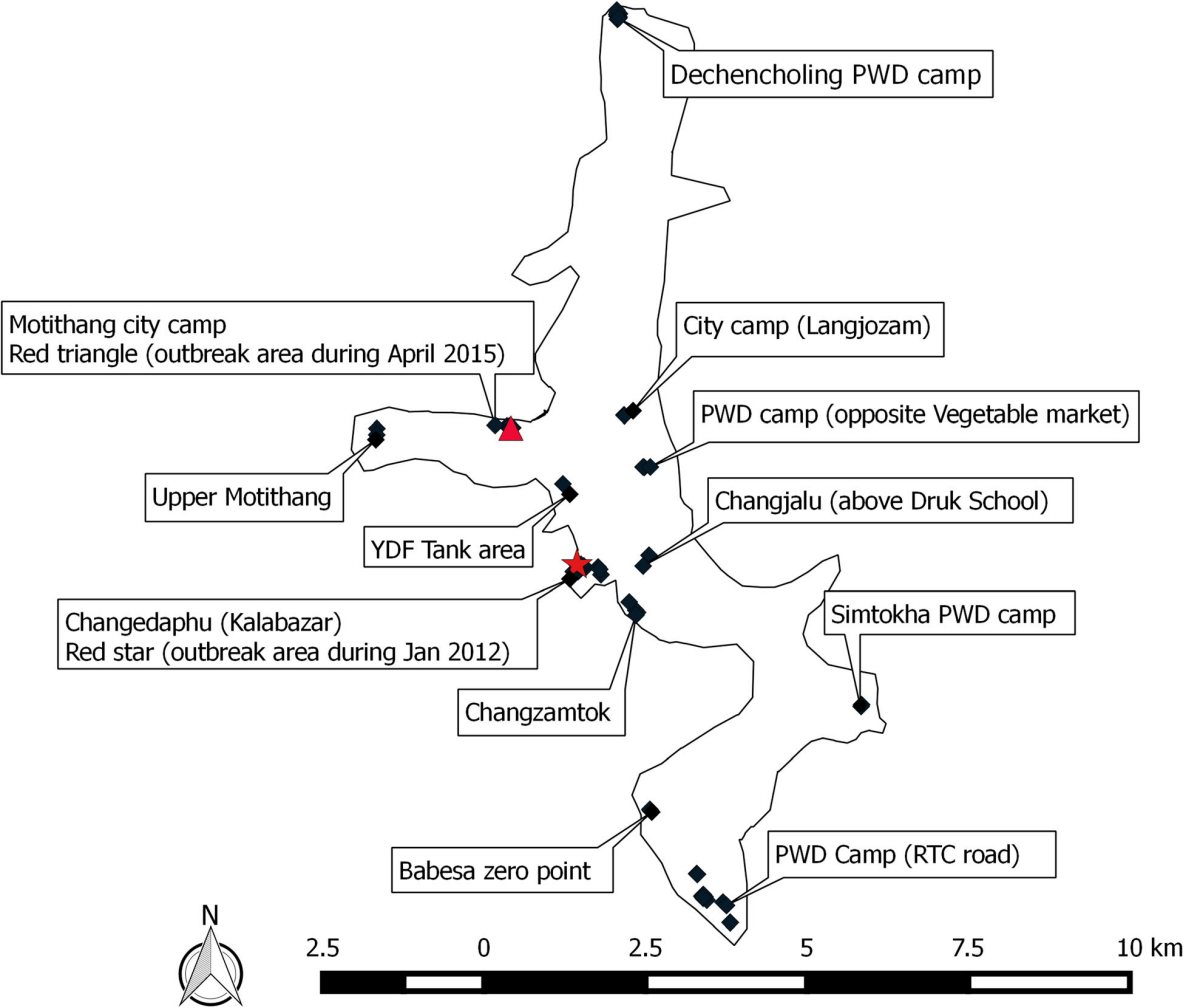
Backyard poultry holding areas in Thimphu City, Bhutan. The location of HPAI outbreak in backyard holding during January 2012 (*star mark*) and April 2015 (*triangle mark*) is shown on the map

This survey was conducted in Thimphu City Area, which is the capital of Bhutan (Fig. 1). The city is located at 27°28′00″N, 89°38′30″E at an altitude of about 2300 m above sea level. Thimphu city covers an area of 26 km^2^ with an estimated population of 93,270. Backyard poultry keeping is practiced in 12 areas within the city inhabited by people who work for Thimphu City Corporation and public work department (PWD) as daily wage laborers’ or on contract system. The first outbreak of avian influenza A (H5N1) was reported in a backyard poultry holding at Changedaphu (Kalabazar) area in January 2012 [16]. The second outbreak also occurred in a backyard holding at Motithang city camp area on 3 April 2015, which is about 3 km away from 2012 outbreak area (Fig. 1). Following this outbreak, we formed a rapid response team (RRT) to implement the containment activities and have identified 12 settlement areas within the city, where poultry birds are reared as backyard free-ranging system (Fig. 1, Table 1).

**Table 1:** Characteristics of backyard poultry farming and the knowledge, attitude and biosecurity practices of poultry owners addressed by the questionnaire

Items	Details
Respondents details	Name, contact detail, place of living, geo-coordinates, gender, occupation, number of people in the household, and approximate monthly family income
Poultry & husbandry characteristics	Number and category of poultry birds kept, breed, source of birds, purpose of keeping poultry by household, number of years of backyard poultry keeping by the household in the city
Biosecurity and management practices	Poultry housing type, location of coop/shed, husbandry practices (intensive/free ranging), contact with wild birds, cleaning & disinfection of poultry house/coop, water bodies near house, foot dip at the entrance to the coop/shed, type of feeds given to the chicken, feeding and watering container, poultry death and disposal system, vaccination of chicken against diseases, notification of poultry death, poultry litter management
Bird flu knowledge and practices, and personnel hygiene	Awareness of bird flu outbreak in the city, source of information, knowledge of bird flu transmission to humans, personnel hygiene (hand washing after handling of chicken and its products, and use of face mask and hand gloves while handling chicken)

### Questionnaire design

A questionnaire consisting of closed questions was designed to collect information on various aspects of backyard chicken keeping and the owners’ knowledge and practices in relation to avian influenza as summarized in Table 1. The questionnaire was piloted with three poultry owners prior to the actual survey and was modified to improve clarity and interpretation.

### Data collection

Owing to the lack of a proper sampling frame, a purposive sampling was used to recruit backyard poultry keepers within the 12 identified settlement. After visiting each settlement, a door-to-door survey was conducted using a rolling sample method in which the first selected household that owned poultry provides information about the next household that owned poultry in the area. In this way, 62 poultry birds owning household (HHs) within the 12 settlement area were selected and interviewed. When the poultry owning HHs was not available during the first visit, we revisited the HHs in the evening after owners returned home after the work, ensuring all the poultry owning HHs were interviewed. One adult person from each selected household/family was interviewed face-to-face. The selected person was informed about the purpose of the survey by explaining that the data collected will be used for understanding the backyard poultry keeping practices and to strengthen backyard poultry biosecurity in the city. All the identified poultry owners (*n* = 62) agreed and consented to be interviewed. Since the questionnaire survey was conducted by the rapid response team as part of an emergency response during the door-to-door surveillance and awareness education campaign during the time of HPAI outbreak in one of city areas, no formal ethical approval was necessary. The interview was carried out from 24 to 27 April 2015. In addition, when a survey team come across any sick birds oropharyngeal and cloacal swabs were collected (*n* = 10) and rapid antigen detection test was performed at the site. The samples were then referred to the laboratory to carry out RT-PCR test to detect H5N1 virus, but none of the samples tested positive to avian influenza A virus and H5N1 virus strain. Awareness education related to poultry bird management, risk of disease spread, biosecurity practices and public health risk of bird flu were provided to the respondents and to the community at the time of interview.

### Data management and analysis

Data was entered into a database developed in Epi Info V.7.1 (http://www.cdc.gov/epiinfo) (CDC, Atlanta, GA, USA). Data cleaning, management and analyses was carried out using Microsoft Excel 2007 (Microsoft Corp., Redmond, WA, USA) and Stata software V.13 (Stata Corp, Texas, USA). The data described in the manuscript can be requested and obtained from the corresponding author.

## Results

### Respondents’ demographic characteristics

Table 2 shows the demographic characteristics of the respondents. Of the 62 participants, 62% (39/62) were female and the majority of the respondents were working for the Thimphu City Corporation and public work department’s road maintenance section in the city as daily wage labourers. The family size of the respondents ranged from 2 to 12 (median 7) and the majority (82%; 51/62) of the family earned an approximate monthly income of Nu. 15,000 (US$ 250).

**Table 2:** Respondents’ demographic, poultry characteristics and purpose of keeping poultry birds

Variables/categories	Number	Percent
Gender of respondent		
Female	39	62.9
Male	23	37.1
Occupation of respondent		
House wife	11	17.74
Work in City Corporation	30	48.39
Work in public road maintenance section	11	17.74
Private work	5	8.06
Others (hospital, forest nursery)	5	8.06
Number of people in the household		
1 to 3	11	17.74
4 to 6	38	61.29
7 to 9	9	14.52
10 to 12	4	6.45
Approximate monthly income		
Up to Nu. 5000	21	33.87
Nu. 5000 to Nu. 15,000	30	48.39
Nu. 15,000 to Nu. 25,000	7	11.29
Above Nu. 25,000	4	6.45
Sources of poultry		
Brought from villages/other areas within the country	12	19.35
Hatched within farm	49	79.03
Government poultry farm	1	1.61
Purposes of keeping poultry birds		
Egg production & family consumption	42	64.52
Egg production & sale	3	4.84
Meat purpose for family	3	4.84
Egg production & meat purpose for family	14	22.58
No. of years of poultry keeping by the HHs		
Less than 1 year	13	20.97
1 to 3 years	14	22.58
3 to 5 years	15	24.19
More than 5 years	20	32.26

### Poultry characteristics and purpose of keeping birds

Sixty two respondents kept a total of 562 local indigenous breed birds (chick: 333, hen: 166, cock: 41) and Hyline brown breed: 22) (Table 3). The main source of the poultry birds was from the hatching of chicks in the households (79%; 49/62) in comparison to purchase of poultry from other places. The majority (67%; 42/62) of the respondents had been keeping poultry for up to 5 years and 32% (20/62) of the owners had been engaged with poultry keeping for more than 5 years. Forty two respondents reported keeping poultry for egg production and family consumption, while 14 respondents kept poultry for both egg production and for meat for family consumption. Only six respondents reported keeping birds for egg production, sale and for meat purposes. In addition 22 respondents also reported keeping poultry birds for sale since local breed fetches higher price (Table 2).

**Table 3:** Number & type of poultry birds reared as backyard poultry in different areas in Thimphu City (April 2015)

Location of risk areas	HHs	Local breed (categories)				Hyline brown breed	Total birds
		Cock	Hen	Chicks	Total		
Changedaphu (Kalabazar)	15	1	28	127	156		156
PWD camp (RTC road)	15	7	28	92	127	2	129
Dechencholing city/PWD camp	9	11	34	21	66		66
Changjalu (above Druk School)	3	7	13	19	39		39
PWD camp (opposite vegetable market)	3	4	10	21	35		35
Motithang city camp	4	1	8	21	30		30
Upper Motithang	2	2	22	1	25	13	38
Changzamtok	3	3	4	16	23		23
City camp (Langjozam)	3	2	5	15	22		22
Babesa zero point area	2	0	8	0	8	7	15
Simtokha (PWD camp)	2	2	4	0	6		6
YDF Tank area	1	1	2	0	3		3
Grand Total	62	41	166	333	540	22	562

### Biosecurity and management practices

The summary of management practices of birds in relation to the biosecurity, disease prevention and control issues is presented in Table 4. Briefly, the poultry birds are housed in a coop constructed with either wooden box (64.5%; 40/62), wire mesh box (12.9%; 8/62), basket (11.3%; 7/62) or coop with wire mesh fencing (11.3%; 7/62) which are either attached to the family house (38.7%; 24/62) or located away from the house (61.3%; 38/62). There is a significant difference (χ^2^ = 32.495, *P*-value = 0.001) between the 12 settlement regarding the location of poultry house in which 17% (12/62) of poultry keepers in Changedaphu (Kalabazar) have the poultry house attached to their house whilst 20.95% (13/62) of the poultry keepers in PWD Camp-RTC road have their poultry house located away from their house.

**Table 4:** Poultry birds management practices in relation to biosecurity practices

Variables/categories	Number	Percent
Type of poultry house/coops		
Basket (made from bamboo)	7	11.3
Wire mesh box coop	8	12.9
Wooden box coop	40	64.5
Wire mesh/wooden box coop with wire mesh fencing	7	11.3
Location of poultry house		
Attached to family house	24	38.71
Outside family house (separate house)	38	61.29
Schedule of poultry house cleaning		
Daily	3	4.92
Weekly	52	83.87
Monthly	7	11.48
Disposal method of poultry litter		
Use as fertilizer in the kitchen garden	54	87.1
Dispose into open area	8	12.9
Sale	0	0
Do the people have access to poultry house		
No	28	45.16
Yes	34	54.84
Do the poultry birds come in contact with wild birds		
No	18	29.03
Yes	44	70.97
Is there water bodies near poultry house/premises		
No	26	41.94
Yes	36	58.06
Type of feed given to poultry birds		
Family food left over	9	14.52
Local feed grains (maize, wheat)	7	11.29
Family food left over & local feed grains	44	70.97
Commercial feed	2	3.23
Have clean container for feeding & watering		
No	29	46.77
Yes	33	53.23
Was there any poultry death during the past 2 weeks		
No	55	88.71
Yes	7	11.29
Way of disposal of dead birds		
Disposal into open area/bush	25	40.32
Burial	37	59.68
Sale	0	0
Consumption	0	0
Have poultry birds been vaccinated against poultry diseases		
No	61	98.39
Yes	1	1.61

All poultry keepers reported cleaning the poultry house daily (4.9%; 3/62), weekly (83.9%; 52/62) or monthly (11.5%; 7/62) but none of the keepers used disinfectant to clean the poultry house or surrounding. The majority (87.1%; 54/62) of the owners used the deep litter produced as fertilizer in the kitchen garden. Some poultry keepers also reported that their poultry houses and birds had access to outside people/visitors (54.8%; 34/62) and contact with wild birds (70.9%; 44/62), particularly feral pigeons since all poultry are reared as free ranging. Six of the 12 settlement locations had a small stream or river near the settlement. The majority of the poultry keepers reported feeding their poultry with left over family food and local grains to supplement the scavenging system, and only 53.2% (33/62) of the keepers used clean containers for feeding and watering birds. Other poultry keepers (46.8%; 29/62) sprayed food/grains into the household surroundings. When asked about any poultry mortality in the backyards, majority (88.7%; 55/62) of the bird keepers reported no unusual mortality during the past 2 week period. The most widely used methods for disposal of dead birds were either burial (59.7%; 37/62) or disposal into open area/bushes (40.3%; 25/62). Of the total 562 birds recorded among the 62 keepers at the time survey, 96% (540/562) of the birds were not vaccinated against poultry diseases since they did not know about the importance of vaccination or even the availability of vaccine. And, only 27.7% (17/62) of the poultry keepers understood how to seek veterinary assistance in the event of any illness in the birds, whilst the rest of the respondents (72.6%; 45/62) were not aware of how to seek assistance.

### Knowledge and practice of personnel hygiene in relation to poultry diseases

When asked whether they had heard of the recent avian influenza H5N1 virus (bird flu) outbreak in one of the city camps in Thimphu, 88.7% (55/62) of the respondents had heard about the outbreak either through livestock surveillance team, news media or friends. More than half (66.1%; 41/62) of the respondents were also aware that bird flu can be transmitted from infected poultry to humans, but how it is transmitted is unknown (Table 5).

**Table 5:** Knowledge about poultry diseases and personal hygiene

Variables/categories	Number	Percent
Heard of bird flu outbreak in Thimphu in the recent weeks		
No	7	11.29
Yes	55	88.71
What were the sources of information^a^		
From disease surveillance team (Livestock)	3	5.45
Television (TV)	14	25.45
Radio news	2	3.64
Print media (Kuensel)	1	1.82
Friends/neighbours	20	36.36
Health officials/clinics	15	27.27
Awareness & knowledge that bird flu can transmit to humans^a^		
No	21	33.87
Yes	41	66.13
Knowledge where to report in case of poultry bird sickness/death		
No	45	72.58
Yes	17	27.42
Where to report the sickness/deaths of poultry^b^		
Livestock Officials	11	64.71
Livestock regulatory authority	4	23.53
City officials	2	11.76

^a^Data based on the total number of person who have heard of bird flu outbreak in Thimphu (*n* = 55) in the previous question

^b^Data based on who have knowledge where to report in case of poultry bird sickness/death (*n* = 17) in the previous question

All the respondents indicated that they understood and practiced hand washing using soap and water after handling poultry and poultry products. When asked whether they used a face-mask while handling poultry or cleaning poultry house, 45 (72.6%) respondents knew the importance of use of face mask but only 22 (48.9%) used one while 23 (51.1%) of the respondents knew of but did not use (practice) face masks. Similarly, 40 (64.1%) respondents knew of the importance of use of hand gloves while handling poultry/products but only 13 (32.5%) practically used gloves while 27 (67.5%) of the respondents knew of but did not use (practice) hand gloves (Table 6). There was no significant (*P* value >0.05) difference between the location, occupation and income level of the respondents with the biosecurity practices and knowledge and practices of personnel hygiene such as use of a facemask and hand gloves while handling poultry and poultry products. There was also no significant (*P* value >0.05) difference between awareness on avian influenza of the poultry keepers with the biosecurity practices.

**Table 6:** Knowledge and practice of hand washing, using face mask and hand gloves while handling poultry & poultry products

Knowledge on the importance of hand wash as well as practice while handling poultry birds			
Knowledge on hand wash	Practice hand wash		Total (percent)
	No (percent)	Yes (percent)	
No	0	0	0
Yes	0	62 (100)	62 (100)
Knowledge on the importance of using facemask as well as practice while handling poultry birds			
Knowledge	Practice (using facemask)		
	No (percent)	Yes (percent)	Total (percent)
No	17	0	17 (27.42)
Yes	23 (51.11)	22 (48.89)	45 (72.58)
Knowledge on the importance of using hand gloves as well as practice while handling poultry birds			
Knowledge	Practice (using hand gloves)		
	No (percent)	Yes (percent)	Total (percent)
No	22	0	22 (35.49)
Yes	27 (67.50)	13 (32.50)	40 (64.52)

## Discussion

To our knowledge this is the first study conducted to explore and assess the biosecurity situation of backyard poultry holdings and the owners’ knowledge and practices in relation to HPAI prevention and control measures among backyard poultry keepers in Thimphu city area. The poultry keeping was found to be a secondary activity, as a means to supplement families’ dietary protein and also generate some additional income for the households. Most birds were of local non-descript breed that either hatched chicks from within the household poultry birds or were bought from other families within the country. However, the result showed that backyard flocks were reared as a free-ranging system where flocks from different households within the settlement scavenged together. During the daytime birds scavenge freely close to the homestead and have access to cheap feed, insects on the ground, water from the drain and waste water accumulation around the houses or stream. Although the nutrient requirement for the chickens may be fulfilled through scavenging feed resources, the birds were also provided feed supplementation such as grains and household family food scraps [17]. But the majority of the poultry keepers have no clean feeder and water container to feed the supplementary feed. Instead, the grains and food scraps are spread around the homestead which also attracts wild birds such as feral pigeons and other birds, providing an avenue for domestic poultry-wild bird interface for disease transmission. Although the risk of HPAI transmission from pigeons and other wild birds into poultry is unclear, there is risk of other avian diseases transmission to both poultry as well as to humans [18].

Regarding poultry housing, the birds are confined in small houses made of wood, wire mesh or are kept in a basket made of bamboo during night time and are released for free-range scavenging during day. The majority of the chicken houses were found to be poor and unhygienic state condition that did not offer adequate protection either from predators and theft or protection against diseases. Therefore, housing systems need to be improved to enhance biosecurity measures. Also the poultry houses were found to be attached to the family house in order to protect them from predators such as stray dogs or from theft. This indicates there is close interaction at the human-poultry bird interface and poses risks for disease transmission. Moreover, biosecurity measures such as disinfection, foot dip, and restriction of visitors have never been implemented in all backyard holdings surveyed in 12 settlements. Since disinfectants are often not easily available in the market it may not be practical to emphasize their use in backyard settings. The cleaning of poultry shed are mostly done on a weekly basis and the wastes products (poultry litter) are used as fertilizer in the kitchen garden. However, the use of untreated poultry manure as fertilizer may pose a risk of infection spread if the birds are infected [11, 19]. In addition, the poultry waste disposal into garden or any land may attract wild birds due to the presence of spilled feed in these wastes thereby infecting wild birds and contributing to long distance transmission [20]. This may be addressed by composting the litter before spread in the garden [11, 19]. Unfortunately, poultry keepers are not aware of and therefore do not practice this measure. In the backyard and resource-poor setting, composting is rarely applied in developing countries [11, 19].

This study also indicates that the majority of the poultry owners are not aware of the existence of veterinary facilities and do not know how to seek veterinary assistance in the event of illness in their chickens. They also do not have any knowledge or awareness of any legal obligation to report any unusual mortality or sickness in their flocks to the veterinary authorities. Another concern is that the majority of poultry keepers dispose of dead birds by burying them in the gardens or dispose into open area/dustbins when the mortality should be reported to the veterinary authority for postmortem examination and investigation. These are inappropriate methods of disposal since any infectious disease outbreaks in poultry, for example, Newcastle disease or HPAI could silently spread within the backyard flocks and act as a perpetual source of infection to other birds in the neighborhood/country as well as pose risk to humans. In backyard poultry farms, sickness or mortality of few number of birds are usually considered as a normal pattern and owners would not normally report these cases. This may be due to limited knowledge of the poultry keepers. The deficiency of knowledge about health problem and relevant regulations such as reporting of any illness or mortality of birds within the flocks/settlement indicate the importance of poultry keepers to have accessible and reliable source of information. Therefore, the veterinary and regulatory agencies should regularly educate the poultry keepers about poultry diseases and biosecurity practices.

In relation to knowledge and awareness of HPAI (bird flu), the majority of the respondents had heard of the recent outbreak of HPAI in a backyard poultry holding in one of the city camps in Thimphu. The study also revealed that poultry keepers are aware of the risk of transmission of disease from poultry to humans. These findings were expected since the current study was conducted shortly after the declaration and announcement of HPAI outbreak in Thimphu in the mass media. Also, the poultry owners have clear memory of the past outbreak containment and awareness program when H5N1 outbreak had occurred in one of the city camps in Thimphu during January 2012.

The findings of this study demonstrate that the poultry keepers are aware of the importance of hand washing with soap and water and undertake washing after handling poultry & poultry products, and after cleaning of poultry shed. This finding is consistent with other studies where hand washing was found to be the best known practice among poultry workers [21, 22]. However, a knowledge gap and practice was found amongst the poultry keepers such as wearing protective hand gloves and face masks while handling poultry or poultry litter. Although some poultry keepers knew of its importance, but were not practiced because the poultry keepers could not afford to procure it for daily use. But there was no variations in the biosecurity practices and personnel hygiene measures between different backyard holdings, occupation, monthly family income and awareness level on avian influenza of the respondents.

## Conclusions

We conclude that the backyard poultry holdings in the study area have very weak biosecurity management practices and the poultry keepers have minimal knowledge and awareness related to the importance of biosecurity measures, veterinary care of the birds and reporting systems, and personnel hygiene. It is therefore important to educate the poultry keepers and improve the housing and management system of poultry farming within the backyard holdings in Thimphu city area in order to prevent future disease outbreak.

## Abbreviations

HH: Households
HPAI: Highly pathogenic avian influenza
PWD: Public work department
RRT: Rapid response team
WHO: World Health Organization
